# Integration of IoT-based healthcare devices within the smart grid architecture model: a conceptual framework with economic and energy assessment for elderly care in Europe

**DOI:** 10.3389/fdgth.2026.1897840

**Published:** 2026-10-15

**Authors:** Azad Aghajani, Marco Ciro Liscio, Lorenzo Scalise, Paolo Sospiro

**Affiliations:** 1 EUAbout, Bruxelles, Belgium; 2 Marche Polytechnic University, DIISM, Ancona, Italy; 3 Marche Polytechnic University, DII, Ancona, Italy; 4 Università Telematica E Campus, DISTA, Novedrate, Italy

**Keywords:** cost-benefit analysis (CBA), digital medical devices, elderly healthcare, internet of things (IoT), Smart Grid Architecture Model (SGAM)

## Abstract

**Introduction:**

This study investigates the integration of IoT-enabled medical devices within the Smart Grid Architecture Model (SGAM) to support elderly home-based care in Europe. The study develops a conceptual SGAM–IoT framework that maps healthcare devices, actors, communication pathways, and functions across the SGAM interoperability layers, domains, and zones, with particular attention to interoperability, data exchange and integration between the home environment, healthcare services, and energy-aware infrastructure.

**Methods:**

The conceptual framework is complemented by a quantitative analytical assessment of the economic and energy implications of five commonly used home-monitoring devices: a pulse oximeter, blood pressure monitor, digital thermometer, smart ECG monitor, and blood glucose monitor. The assessment relies on secondary European healthcare data, literature, manufacturer information, and market-based estimates rather than primary empirical measurements or experimental deployment. Economic feasibility is examined through Benefit-Cost Ratio (BCR), Net Present Value (NPV), and a three-scenario sensitivity analysis, while electricity consumption is estimated from rated device power and assumed operating time.

**Results:**

Under the reference-scenario assumptions, the analysis produces a BCR of 2.75 and a positive NPV, while the sensitivity analysis indicates that the economic result remains positive under conservative assumptions. The revised energy assessment estimates a combined electricity consumption of approximately 0.12 kWh per month, confirming that the direct energy demand of the selected devices is marginal.

**Discussion:**

These findings suggest that the main value of SGAM in this context lies not in device-level energy savings, but in providing a structured framework for interoperability, communication, data exchange, and integration with wider home-healthcare infrastructure. Potential benefits for continuity of care, autonomy and elderly well-being are discussed as expected implications requiring future empirical validation.

## 1 Introduction

The shift toward digital health and complex modern living has led individuals to increasingly rely on smart medical devices for personal health management. Globally, the population of 65 years and older is expected to overtake the population of children by the late 2070s, with 2.2 billion people at this age (1). Older people represent solid and relevant contributors to the community wealth, and their wellness has positive impacts on the whole family, like peace of mind and self-confidence (2).

Zhu et al. meta-analysis revealed that a significant percentage of older adults worldwide suffer from multimorbidity and chronic diseases such as cardiovascular issues, diabetes, and movement disability, with a prevalence of 46% (3). There are a myriad of mental diseases linking to aging like cognitive impairment (dementia, Alzheimer, schizophrenia), and depression. Elderly people frequently encounter loneliness and social isolation, which impact on their physical and psychological well-being (4).

Digital healthcare devices are produced to meet the well-being needs of people. They turned into daily usable devices, measuring and showing the various values of vital signs and other health indicators (5). There are different types of these devices including wearable devices, remote monitoring systems, telemedicine devices, and implantable devices employing the-state-of-art technologies to ameliorate wellness among people (6). The concept of Internet of Things (IoT) refers to a physically linked network between things that are integrated into various systems and devices to send data via the web (7). Many studies and models, describing the IoT and smart grid technologies (8, 9, 33), while the accumulated models that adapt digital medical devices are limited. Milon et al. (10) provided a clear overview of IoT technology by briefly demonstrating the most widely used IoT device features, architectures, and protocols.

Moosavi et al. (11) introduced a distributed smart e-health gateway based on a secure and efficient authentication (SEA) framework which provides an efficient and secure way to oversee authentication and authorization in IoT-based healthcare systems. The Moon et al. (12) project aimed to make a pulse oximeter that works with the Internet of Things in medical settings that use edge computing. Saleem et al. discussed the SGAM as a reference architecture designed to provide an architectural perspective on the use cases of the Smart Grid. It was created by the EU Mandate M/490s working group on reference architecture (13). Ganesan et al. (14) created a smart Human Activity Recognition (HAR) system for identifying the physical activity of senior citizens, improving the whole system's dependability, and suggested utilizing a Smart Grid (SG) monitoring system to track the wearable device's operating state. Three levels make up this architecture, including fog, cloud, and things layers (15).

This research aims to adapt the SGAM architecture to the IoT-enabled home-healthcare context and to provide an analytical assessment of its economic and energy implications for elderly care in Europe. It also discusses the potential implications of the proposed framework for remote monitoring and continuity of care.

## 2 Materials and methods

### 2.1 Overview

The age distribution of society is changing quickly, and Europeans are alive longer than at any time before. As a result of demographic aging, the proportion of working-age individuals is declining while the proportion of elderly individuals is rising (16). The proportion of the adult population struggling with daily life and basic activities dilemma like eating, bathing, and dressing, rises with age. One of the principal reasons behind this pattern is the high share of older people who suffer from physical and sensory functional limitations, impacting their vision, hearing, mobility, communication, or ability to remember (17). All parameters used in the analytical components are derived from secondary data sources, literature, and market-based estimates. The objective is to provide a structured framework and indicative evaluation rather than empirical validation.

### 2.2 Study design and analytical procedure

The study adopts a structured analytical methodology to evaluate the integration of IoT-enabled healthcare devices within the Smart Grid Architecture Model (SGAM). It follows a structured analytical methodology combining system architecture modelling with quantitative evaluation. The research procedure consists of the several steps including devices selection, SGAM mapping, parameters definition, analytical modelling, and assumptions.

Five commonly used home healthcare devices were selected based on measuring vital signs and relevance to elderly care: pulse oximeter, blood pressure monitor, digital thermometer, smart ECG device, and blood glucose monitor. Each device was mapped into the SGAM framework across Five interoperability layers (business, function, information, communication, components), Domains (distribution, customer premises), and Zones (process, field, station, operation, enterprise, and market). Economic and energy parameters were defined as two key analytical parameters. Economic parameters include device cost, maintenance expenses, and healthcare saving. Energy parameters focus on power consumption, and usage duration. A five-year evaluation period was selected to reflect a realistic lifecycle of the devices.

The study applies several analytical models to evaluate system performance and feasibility. The Benefit-Cost Ratio (BCR) is used to assess economic efficiency by comparing total benefits with total costs over the defined period. The Net Present Value (NPV) is calculated to determine the long-term financial viability of the investment, taking into account the time value of money. Additionally, the energy consumption model is applied to estimate the total energy usage of the selected devices and to assess their impact on household electricity demand.

The analysis includes a set of consistent and clear assumptions. Device specifications were obtained from existing literature and manufacturer data. Healthcare cost estimates were based on European averages, and a fixed discount rate of 5% was applied to account for the time value of money and uncertainty about future benefits.

Accordingly, the study comprises two complementary components: (i) conceptual adaptation and mapping of SGAM to the home-healthcare use case, and (ii) a quantitative analytical assessment of the economic and energy implications of the selected device configuration.

### 2.3 Remote monitoring and features of healthcare devices

Remote healthcare monitoring technologies provide an alternative to in-person clinical visit by facilitating ongoing observation of patient health condition at home. These systems typically rely on non-invasive wearable or portable devices that measure physiological parameters and transmit data to healthcare providers in real time (18). The physical hardware sensors collect physiological indicators such as blood pressure (BP), respiration rate (RR), body temperature, heart rate (HR), oxygen saturation (SpO2), and electrocardiogram (ECG) (19). Wearable sensors are designed to operate with minimum disruption to daily activities which enable early health change detection (20). We evaluated the devices used to measure the aforementioned vital signs. The selection process for these health devices supporting the aging population depended on the suitability for primary care monitoring, their efficacy in the early detection of chronic and acute conditions, and their accessibility in the European market. For this purpose, five commonly used and widely available home-monitoring devices were selected, including pulse oximeters, blood pressure monitors, digital thermometers, smart ECG devices, and blood glucose monitors (21–23). These non-invasive, portable, and user-friendly devices facilitate easy integration into IoT healthcare systems, particularly benefiting elderly users with limited technical skills. Wireless connectivity via Bluetooth or Wi-Fi enables data transmission to smartphones, home gateways, or cloud-based platforms for real-time remote monitoring. Although the analysis is concerned with these specific devices, the findings are not limited to them. The results can be extended to other IoT-based healthcare devices which have similar features. However, we can say that more advanced or resource-heavy devices might show different performance, cost, and energy consumption patterns and these aspects should be investigated in future research.

In healthcare, IoT is applied to create a network of apps and devices that connect patients and medical professionals. These systems diagnose, track, and store important health data. There are several primary areas where IoT and wearable devices can help monitor and support older adults (24). IoT systems are implemented using a variety of technologies, including the Internet and mobile networks, wireless sensor networks (WSN), infrared sensors, radio frequency identification (RFID), and global positioning systems (GPS) (25).

The physical layer's principal job is to detect the physical properties of the objects in the environment. Radio frequency identification (RFID) and global positioning system (GPS) are just a few of the sensing technologies it uses (26). The data link layer handles packet boundaries, frame synchronization, address management, fault detection, and collision avoidance. The network layer provides data routing through the network, using devices like switches and routers. The transport layer works with the application layer to ensure error-free data transmission, simultaneously handling data reliability, order, and congestion (27). Most of the technology's potential is realized at the application layer, which acts as the front end of the IoT architecture. One essential precondition for the development of IoT is achieving low power consumption (28).

### 2.4 Conceptual smart grid architecture model (SGAM)

Energy networks known as smart grids can automatically track energy flows and adapt to variations in the supply and demand for energy. Smart grid deployment is one of the three key subject areas of the Trans-European Networks for Energy, which aims to complete the European energy market, assist in integrating renewable energy, and give consumers more control over how much energy they use (29). The smart grid conceptual model unveiled by the National Institute of Standards and Technology (NIST) offers a high-level framework for the smart grid. It identifies seven high-level domains and illustrates all the electricity and communications flows that connect each of them as well as their relationships. This three-dimensional model (Figure 1) combines the five interoperability layers (business, function, information, communication, and component) with the two smart grid plane dimensions: domains (which cover the entire electrical energy conversion chain, including bulk generation, transmission, distribution, distributed energy resource, and customers premises) and zones (which represent the hierarchical levels of power system management: process, field, station, operation, enterprise, and market) (25, 30). The SGAM is designed initially to model energy systems. The Market zone of the original SGAM was not included in the proposed healthcare adaptation because the framework focuses on elderly home-based healthcare services rather than electricity-market interactions. But thanks to its layered structure, it can easily be adapted and simplified to other fields, like healthcare. By aligning healthcare elements with the SGAM layers, we can investigate system's behaviour in a much more structured and systematic way. The study makes use of SGAM as a conceptual framework for modelling the integration of IoT-enabled healthcare devices into a system which facilitates the identification of integration points, communication pathways, and functions between different sectors. This approach enhances the understanding of how medical devices operate within an adapted system that provide a base for conceptual and quantitative analysis.

**Figure 1:**
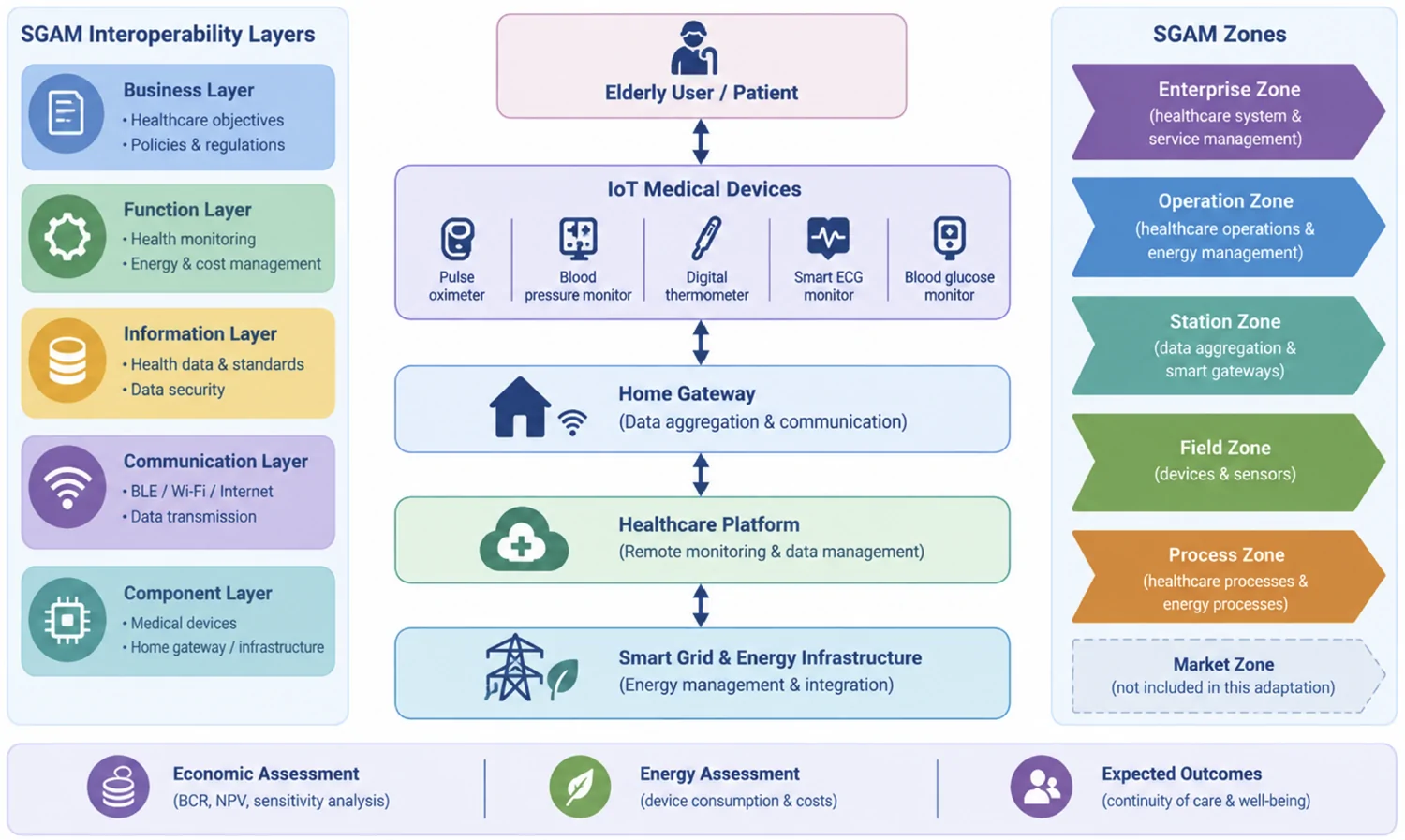
A conceptual framework of IoT-based healthcare devices within SGAM. Own elaboration based on (30).

### 2.5 SGAM-IoT framework for digital health devices

While healthcare devices have their own performance and usage, some common characteristics allow us to utilize the SGAM framework to evaluate the benefits of the device for elderly people from various perspectives, including energy consumption, cost-benefit analysis, and overall impact on well-being. Hence, a structure that aligns with the different layers and domains of the SGAM based on IoT, considering all effective aspects, can be achieved (Figure 2).

**Figure 2:**
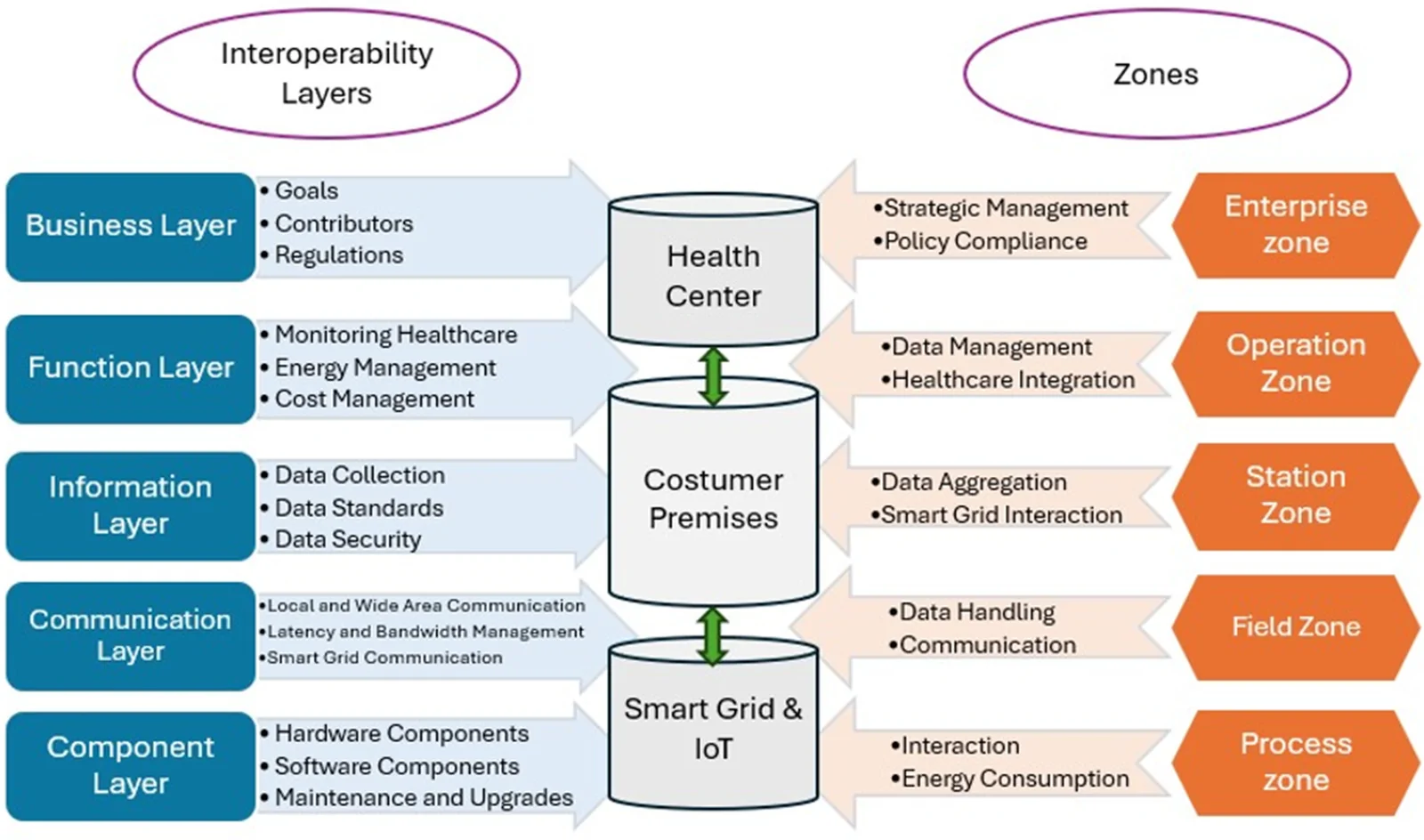
Adaptation of the Smart Grid Architecture Model (SGAM) for IoT-enabled elderly home healthcare. Own elaboration based on (25, 30).

#### 2.5.1 Business layer for healthcare devices

The business layer defines the system's goals, contributors, and regulations. The purpose of the goals is to specify the main objectives of the project. Its main priorities are Health monitoring, cost reduction, and energy saving. Continuous monitoring of physiological parameters is performed to ensure early detection of potential health issues in the elderly. Cost reduction decreases healthcare costs by enabling early detection of issues and reducing the need for frequent physician visits in health centres. Energy saving optimizes the energy consumption of the monitor or device within a smart grid environment to extend battery life and reduce operating costs. Contributors are those who take advantage of using these devices somehow like senior people. Regulatory compliance ensures that the device complies with EU regulations, General Data Protection Regulation (GDPR) law, CE marking, and medical device standards.

#### 2.5.2 Function layer for healthcare devices

The Function layer guarantees the capabilities of the device, which are in line with the general objectives of older adults' health. It covers three areas: healthcare monitoring, cost management, and energy management. Healthcare monitoring is essential for monitoring the physiological health of older people. Most of the selected Medical Devices (MD) measure and monitor one or some of the critical signs. Energy management covers three sectors, including power management, battery optimization, and smart grid integration. When the device is not actively collecting or sending data, it enters power-saving modes. Battery life is increased, and energy expenses are decreased by this feature, which lowers energy use when the device is inactive. The lifespan of the monitor's battery depends on effective power management, which guarantees that the device is usable for extended periods. This is especially critical for older patients who might require continuous monitoring. The devices use energy-efficient components and algorithms to optimize battery usage. This function ensures that the devices consume the least amount of power necessary for their operation, extending the time between charges. Keeping the battery working well is important, especially for older users who might not have easy access to charging. Smart grid integration enables the device to communicate with the smart grid to optimize energy use based on real-time energy availability and cost. The device can adjust its power consumption in response to signals from the smart grid, which helps to reduce energy costs. Being connected to the smart grid ensures the device runs efficiently, especially where energy prices vary, which can save money for users.

#### 2.5.3 Information layer for healthcare devices

Data collection as information layer addresses the demand for reliable data storage solutions for handling vast amounts of health data. It focuses on how the monitor collects and securely stores patient data such as blood pressure, oxygen levels, and ECG. By using secure communication methods, encryption, and adherence to data privacy rules, data security makes sure that patient information is shielded from unwanted access. This is especially important because health data in Europe is considered very private. The IEC 61850 standard helps share energy consumption data to the smart grid. Devices that capture data are assured to be interoperable with various healthcare systems, including hospital information systems and electronic health records (EHRs), thanks to data interoperability. Data analytics entails using data analytics methods to process and analyse health data, allowing health care providers to detect patterns, predict potential health issues, and make informed decisions.

#### 2.5.4 Communication layer for healthcare devices

The communication layer focuses on the network and communication rules that let devices, gateways, and the smart grid share data safely and efficiently. Local communication describes how the device communicates with neighbouring devices, such as smartphones, Bluetooth, Wi-Fi, or other wireless protocols. This is crucial for real-time data transmission at the patient's home. Broader networks, such as the internet, are used for wide-area communication between monitor and cloud-based systems or healthcare providers. Network security makes sure that all communications are safe from online attacks and that patient data is kept private. This calls for frequent security upgrades and the use of secure communication channels. Vital indicators are transmitted with the least amount of delay. Using edge computing helps reduce the amount of data sent over the network. By lowering latency and bandwidth consumption, this can improve the effectiveness of real-time monitoring. It also uses algorithms to focus on the most important data, sending only what is needed to keep network traffic from getting too busy. The device can receive signals from the smart grid indicating peak energy usage times or energy price fluctuations. In response, the device can adjust its energy consumption (e.g., entering a low-power mode) to reduce costs and manage power more efficiently. For contribution to the overall energy management strategy of a smart home or healthcare facility, a device can share its energy usage and status with the smart grid. The IEEE 11073 standard is made for personal health devices to connect smoothly with other medical tools and systems. Following IEEE 11073 means the monitor's data can fit into bigger health management systems.

#### 2.5.5 Component layer for healthcare devices

Component layer includes all the hardware and software components that make up the device, along with their interconnections and functionalities. This layer provides a comprehensive overview of the tangible elements that work together to deliver healthcare services. Hardware components include the devices' sensors, CPUs, microcontrollers, and internal batteries. There are various elements that cooperate effectively to gather and send health information. The operating system, application software, and firmware that operate on the display are all considered software components. The software is reliable and able to manage the communication and data processing duties that the device requires. IoT gateways in smart homes manage connectivity with the cloud, control local data processing, and gather and aggregate data from monitors. Maintenance and upgrades involve the ongoing maintenance of the device's components, including regular software updates and hardware checks to help the device remain functional and up to date over time.

#### 2.5.6 Zones and domains for healthcare devices

Domains in SGAM include generation, transmission (wide area network), distribution (neighbourhood area network), distributed energy resources (DER), and customer premises. The customer premises domain relates to the end-users or consumers in energy systems. In the context of healthcare, an elderly individual's home, where the portable medical devices are used, determines the domain. The home area network (HAN) refers to the network within a household that connects various devices which enable communication and sharing data. It connects various health monitoring devices within the patient's home to a central hub, such as a home gateway, which then connects to external networks (like a health centre system or cloud services).

Zones are a necessary component of SGAM because they help to organize the complex interactions and layers of a smart grid into manageable segments. The zones in the SGAM framework represent various levels of functionality. Each zone defines a specific layer of abstraction within the system, ensuring that all aspects of operation and management are considered. Process zone, field zone, station zone, operation zone, and enterprise zone are five levels which integrated with domain as indicated in Figure 2.

To make the adaptation of SGAM to the elderly home-healthcare use case more explicit, Table 1 summarizes the correspondence between the five SGAM interoperability layers and the principal healthcare functions, actors/components, data exchanges and communication standards considered in the proposed framework. The mapping should be interpreted as an adaptation of the SGAM-IoT structure to the healthcare use case rather than as a modification of the original SGAM architecture.

**Table 1:** Mapping of the SGAM-ioT healthcare devices framework.

SGAM layer	Healthcare element/Function	Main actor/Component	Data/Communication	Relevant standard
Business	Definition of healthcare objectives and system-level requirements, including continuous health monitoring, cost management, and energy-aware operation	Elderly user/patient; healthcare provider/health centre; system/service operators	High-level requirements concerning healthcare services, operational objectives, and regulatory constraints	No specific technical standard assigned at this layer
Function	Health monitoring; collection of vital-sign measurements; remote monitoring; cost management; energy management	Medical devices; home gateway; healthcare monitoring system; smart-grid services	Functional exchange between monitoring devices, home infrastructure, and healthcare services	Functional requirements implemented through the standards used at the information and communication layers
Information	Collection, organisation, storage and secure management of health and device-related information; management of energy-consumption information	Medical devices; home gateway; cloud/data platform; health-centre information system	Vital-sign data, device status, energy-consumption data, and information required for remote monitoring	IEC 61850 for exchange of energy-related information within the smart-grid environment
Communication	Communication between healthcare devices, the home gateway, cloud/healthcare systems, and smart-grid infrastructure	IoT-enabled medical devices; smartphone/home gateway; wireless network; health centre; cloud platform	Local device-to-gateway communication through Bluetooth/Wi-Fi and wider communication between the home, external healthcare services, and digital platforms	IEEE 11073 for interoperability of personal health devices; Bluetooth/Wi-Fi communication protocols
Component	Physical and software elements enabling sensing, processing, connectivity, and system integration	Pulse oximeter; blood-pressure monitor; digital thermometer; smart ECG; blood-glucose monitor; gateway; IoT/cloud platform; smart-grid interface	Physical acquisition of measurements, local processing, transmission, and connection to external systems	Device and gateway interfaces implementing IEEE 11073 and IEC 61850 where applicable

Own elaboration.

Regarding the SGAM plane, the healthcare application is primarily located in the customer-premises domain, represented by the elderly user's home and its Home Area Network (HAN). The portable healthcare devices constitute the primary sensing components and communicate with a home gateway, which connects the local environment to external healthcare systems and cloud-based services. The interaction with the smart-grid infrastructure enables device and energy-related information to be incorporated within the wider digital home environment. Across the SGAM zones, device measurement and interaction occur mainly at the process and field levels, while data aggregation and gateway functions correspond to the station level, healthcare monitoring and data management to the operation level, and strategic management and policy-related functions to the enterprise level. This mapping therefore establishes a data flow from the medical device and patient environment, through the home gateway and digital platform, towards healthcare providers and, where relevant, smart-grid services.

The relevance of SGAM in this healthcare context does not primarily derive from the electricity consumption of the individual medical devices, which remains very limited. Rather, SGAM provides an architectural framework through which heterogeneous healthcare devices, home gateways, communication networks, cloud-based services, healthcare providers, and the energy-aware home environment can be represented within a common structure. This allows requirements related to interoperability, reliable data exchange, service continuity, and communication between the different system components to be considered and coordinated across the SGAM layers. Energy management therefore represents one dimension of the framework, rather than its principal justification in the proposed healthcare application.

#### 2.5.7 The schematic framework of SGAM

Using the SGAM framework, this study assesses the medical devices across five layers: business, function, information, communication, and component. These layers are mapped to corresponding zones, allowing for a holistic analysis that considers the device's operation from the physical process level to high-level enterprise management. Therefore, turning the adapted SGAM framework into a practical and applied framework, which considers all indicated elements, can be accurate and mostly reliable. Figure 3 depicts the detailed oriented schematic framework of SGAM to analyse cost-benefits, energy consumption, and health monitoring of medical devices efficiently.

**Figure 3:**
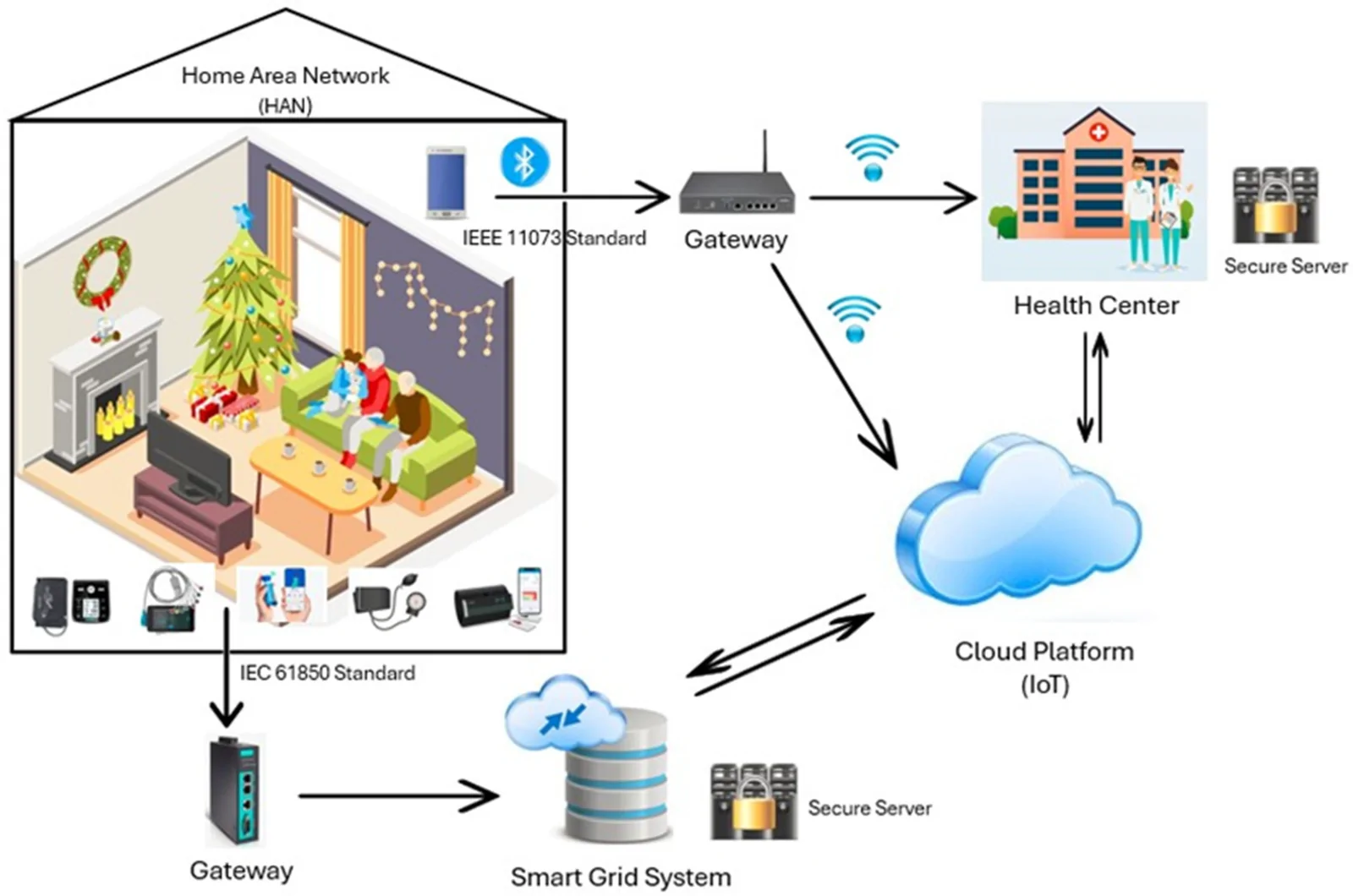
Oriented schematic framework of SGAM. Own elaboration.

## 3 Results

The function layer of the SGAM framework deals with the monitoring of vital signs in older adults, obtained from digital healthcare device measurements. Moreover, if they spend money on devices to monitor vital signs that are available to most households in Europe, the objective of preventing diseases will be true. Therefore, only the most used devices, like pulse oximetry, blood pressure monitoring, digital electronic thermometer, smart ECG monitoring, and blood glucose level monitoring, were considered for calculation in this research.

### 3.1 Cost-benefit analysis

Cost-benefit analysis (CBA) is a method to estimate some important financial indices. It assesses the costs and benefits of using medical devices by various indicators to reveal whether the financial advantages outweigh the costs or not. The CBA has two significant indicators: benefit-cost ratio (BCR) and net present value (NPV), providing a detailed analysis through a systematic assessment. These parameters quantify and compare costs and benefits in a long-term period. The BCR computation is based on comparing total costs and benefits, and if the ratio is greater than 1, meaning the project is feasible economically, then the advantages of utilizing the medical devices outweigh the expenses.

The NPV indicates the crucial importance of investment in smart medical devices over time and helps avoid a loss for a long time. It uses a discount rate to find the present value of expected cash inflows and outflows over a period. The positive NPV boosts the stakeholders' trust in implementing the project in the long term. The costs include buying and using the devices that promote initial investment, maintenance, and operating expenses. Regarding benefits, early detection of health problems and reductions in hospital and physician visits result in monetary and health savings contributing directly to the well-being system.

#### 3.1.1 Calculation of benefit-cost ratio (BCR)

The benefit-cost ratio (BCR) is a financial tool that helps evaluate the economic value of an investment by comparing expected benefits with the costs involved. The BCR assesses the cost-effectiveness of the devices for elderly individuals over five years. Under the reference-scenario assumptions, the resulting BCR of 2.75 indicates €2.75 of assumed economic benefits for each euro of estimated cost. All calculations are regarding aggregated prices for all the devices below, meaning that if a senior buys these devices, all vital signs can be evaluated and meet the needs of primary health care services (PHC), which is identified by the World Health Organization (WHO) as a prevention of diseases protocol and program. The BCR evaluates whether investing in digital healthcare devices is financially worthwhile over five years or not. It reflects the continuous expenses of keeping the devices functional over their expected lifetime. The expected lifetime of the mentioned medical devices is usually 7 years or more; however, for a secure estimation, it is assumed that each device has a 5-year lifespan. Table 2 shows the cost table for each device. Operational costs are the sum of the new battery and maintenance costs.

**Table 2:** Costs table summary for 5 commonly home-used devices.

Devices list	Purchase price (€)	Battery costs (€) - annually	Maintenance costs (€) - annually	Operational costs (€) - annually	Operational costs (€) - 5 years	Total 5 years costs
Pulse oximetry	40	4	8	12	60	100
Blood pressure monitoring	60	6	12	18	90	150
Digital thermometer	20	2	4	6	30	50
Ecg monitoring	80	8	16	24	120	200
Blood glucose monitoring	40	4	8	12	60	100
Total	**240**	**24**	**48**	**72**	**360**	**600**

Healthcare expenditure and reimbursement arrangements vary substantially across European countries. Therefore, the economic assessment developed in this study does not aim to estimate an observed or universally applicable saving for elderly users. Instead, EU-level healthcare expenditure data (31) are used as a reference for defining an indicative annual benefit scenario. Based on this reference and the assumptions reported below, an annual benefit of €330 is adopted in the baseline scenario to represent the potential combined economic effect of earlier detection and reduced use of in-person healthcare services. This value should therefore be interpreted as an analytical assumption used for the cost-benefit assessment rather than as an empirically observed saving. The assumed annual benefit is divided into two broad categories for analytical purposes: potential benefits associated with earlier detection of health conditions and potential benefits associated with reduced use of in-person healthcare services. Tables 3, 4 present the indicative values adopted to construct the reference scenario. These values are not intended to represent directly observed savings for individual patients or healthcare systems but provide a transparent basis for economic modelling.

**Table 3:** Indicative benefit assumptions associated with earlier detection used in the reference scenario.

Early detection	Description	Saving amounts per year (€)
Travel costs	Fewer appointments, less time spent on transportation	40–50
Specialist referral	Reduce the need for a specialist doctor	30–40
Unnecessary treatments	Prevent unnecessary medications and reduce surgeries in the hospital	80–90

Authors’ elaboration using EU healthcare expenditure data (31) as a reference.

**Table 4:** Indicative benefit assumptions associated with reduced use of in-person healthcare services used in the reference scenario.

Reduction in hospital visits	Description	Saving amounts per year (€)
Hospital staying	Minimize the hospitalization	90–100
Routine examination	Reduce frequent check-ups in the health centre	20–30
Insurance payment	Decrease the partial health insurance cost	70–80

Authors’ elaboration using EU healthcare expenditure data (31) as a reference.

The second component of the reference scenario represents potential benefits associated with a lower use of hospital-based and routine in-person healthcare services. For consistency with the conservative orientation of the baseline assessment, the lower values of the ranges reported in Tables 3, 4 are used to construct the assumed annual benefit.

In other words, these are the returns or gains from using the devices regularly. Figure 4 represents the benefit breakdown of devices into a donut chart. Combining the two categories, the reference scenario assumes an annual benefit of €330. Over the five-year analytical period, this corresponds to a total undiscounted benefit of €1,650. This amount represents a scenario assumption for assessing the potential economic feasibility of the proposed configuration and should not be interpreted as an observed healthcare saving. The analysis does not include connectivity charges, healthcare-professional monitoring time, digital-platform costs, or wider infrastructure investments and should therefore be interpreted as a simplified device-level reference scenario rather than a full societal cost-benefit analysis.

**Figure 4:**
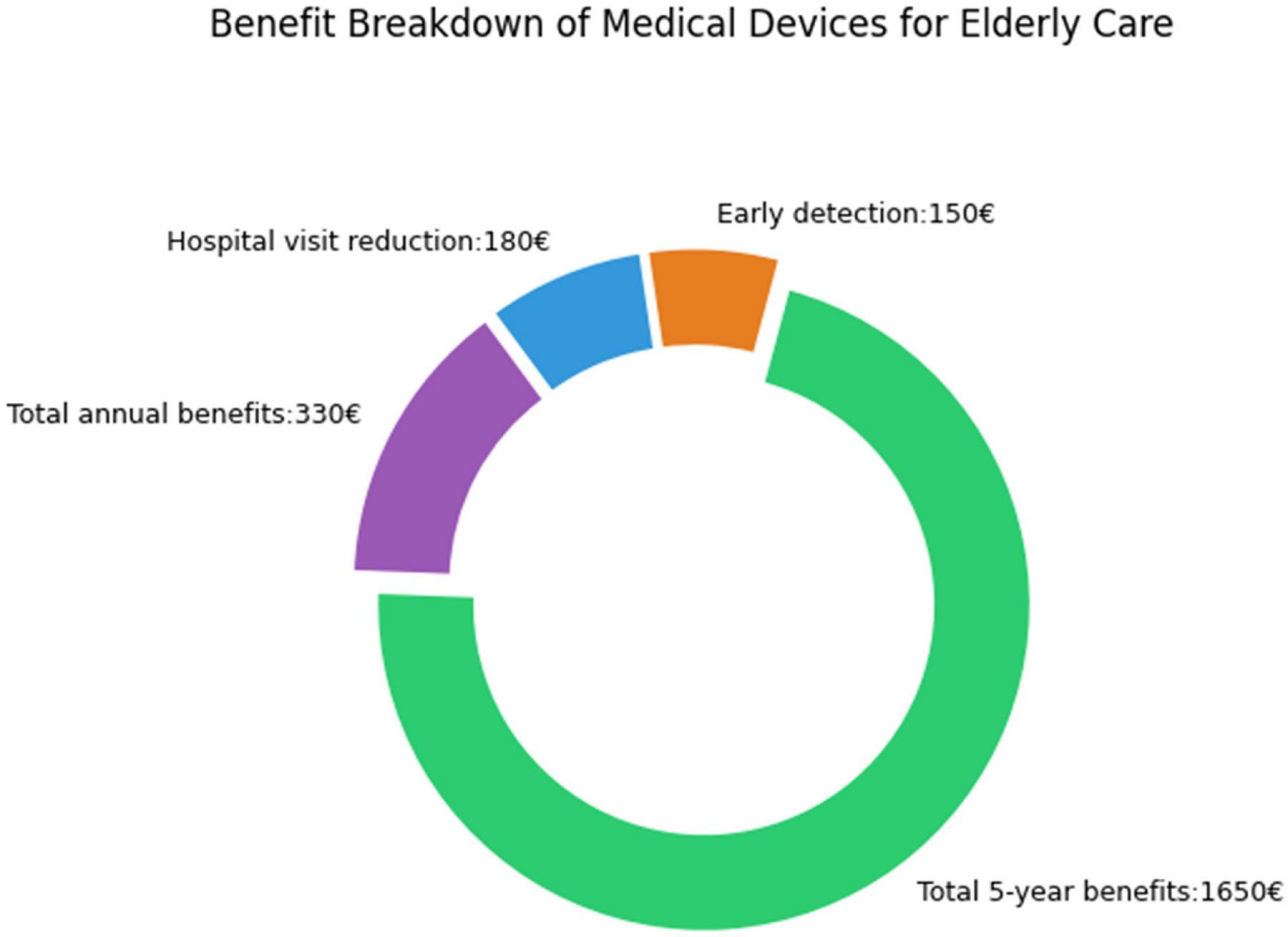
Benefits proportion for using the medical devices.

The BCR formula (1) was performed by the ratio of total benefits over total costs for 5 years as follows:

$$\mathrm{BCR}\;\mathrm{formula}\;=\;\frac{\mathrm{Total}\;\mathrm{Benefits}}{\mathrm{Total}\;\mathrm{Costs}}\;=\;\frac{1650}{600}\;=\;2.75$$ (1)

Under the assumptions adopted in the reference scenario, the resulting BCR is 2.75, meaning that each euro of estimated cost is associated with €2.75 of assumed gross benefits over the five-year analytical period. A BCR greater than one indicates that the proposed configuration is economically favourable under the assumptions of this reference scenario. However, the result is sensitive to the assumed healthcare benefits and cost parameters and should not be interpreted as an empirically established return. The comparison between total costs and total benefits is exhibited in Figure 5.

**Figure 5:**
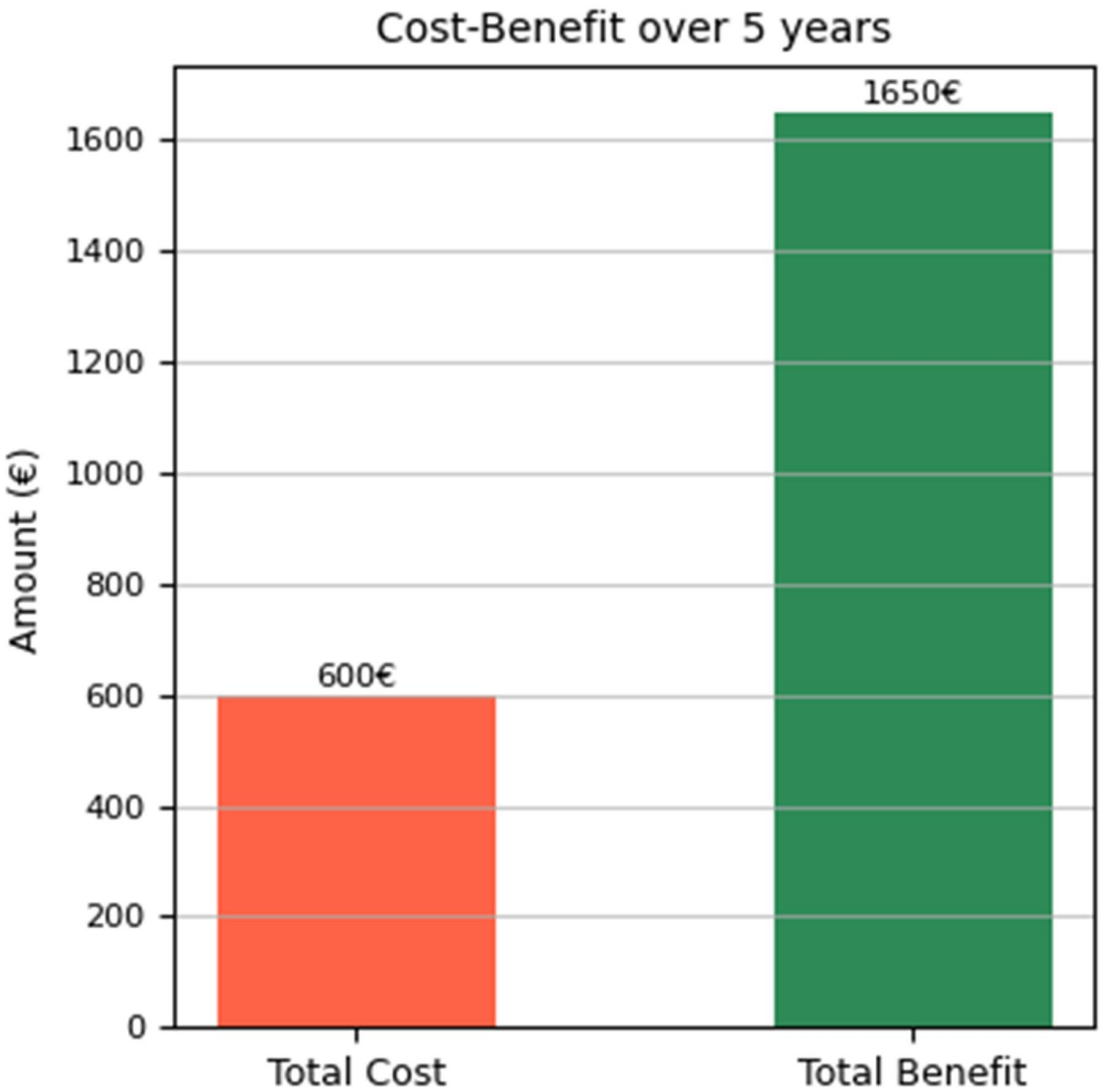
Total costs and benefits bar chart based on the calculations.

Because healthcare costs, reimbursement mechanisms, service-use patterns, and patient contributions differ across European countries, the resulting BCR is not directly generalizable to individual national healthcare systems.

#### 3.1.2 Calculation of net present value (NPV)

Net Present Value (NPV) is a financial metric used to evaluate the profitability of purchasing by calculating the present value of expected future cash flows, considering the time value of money. The result of the NPV formula (2) tells us the net gain or loss in today's money. A positive NPV means that the present value of the benefits is higher than the costs, and a negative NPV indicates that the investment is not profitable. In this study, a 5% discount rate was chosen to account for the trade-off between present and future benefits and the uncertainty in future asset values. The formula is:

$$\mathrm{NPV}\;\mathrm{formula}\;=\;\;\Sigma \frac{B(i)-C(i)}{{\left(1+r\right)}^{i}}$$ (2)

The positive NPV indicates that, under the assumptions adopted in the reference scenario, the present value of the assumed benefits exceeds the estimated costs over the five-year analytical period. The NPV from year zero to year five is shown in a line graph in Figure 6. The initial investment starts at a negative amount (−240€), becomes positive in the first year with 6€, and increases each year. By the fifth year, the cumulative NPV reaches 876€, confirming a strong long-term gain.

**Figure 6:**
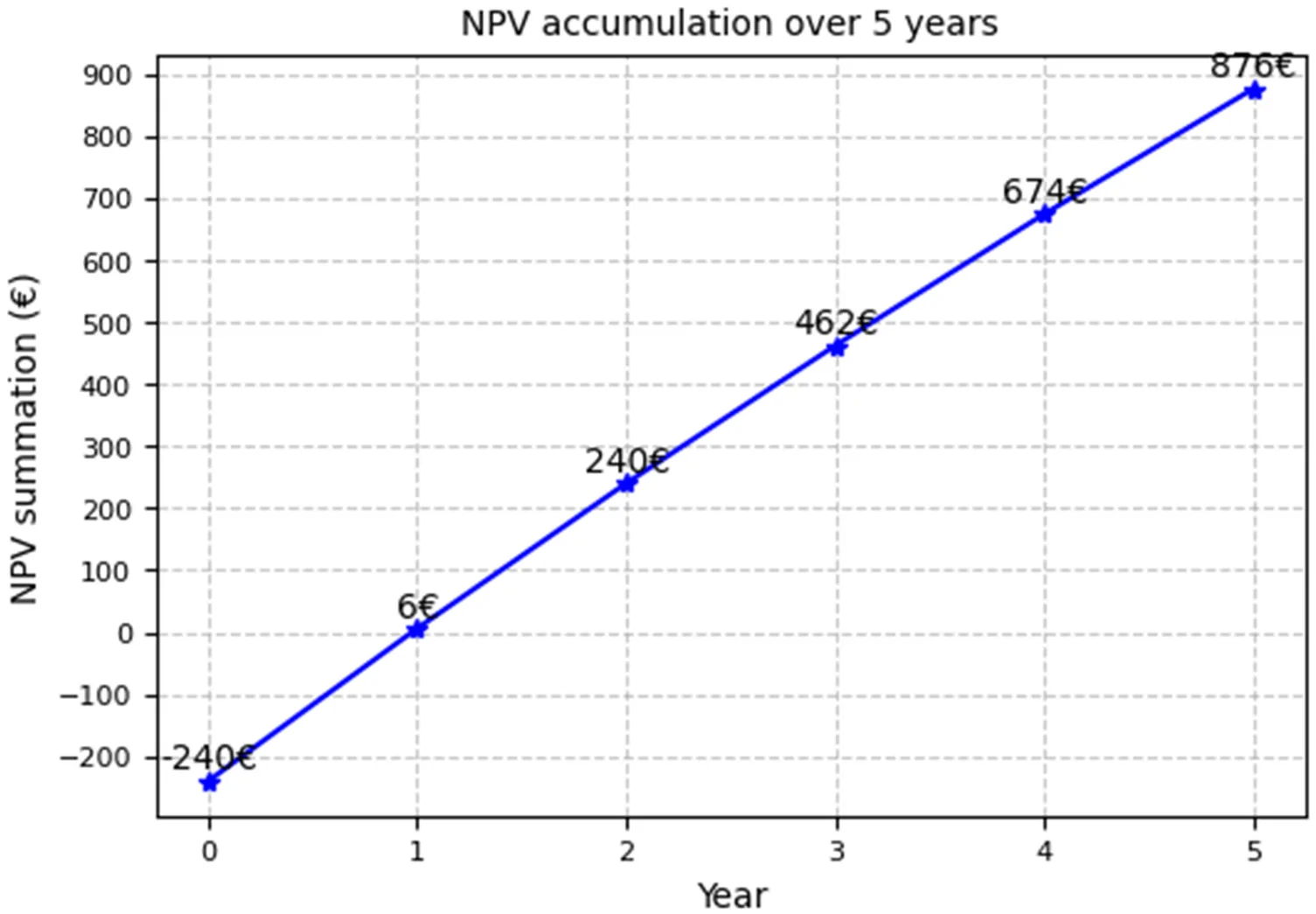
Net present value graph of 5 years of accumulated values. Own elaboration.

#### 3.1.3 Sensitivity analysis

Since the economic results depend on assumptions concerning device costs, operating expenses, expected healthcare benefits and the discount rate, a simple scenario-based sensitivity analysis was conducted. Three scenarios were considered: conservative, reference and optimistic. The five-year analytical horizon was maintained across all scenarios to ensure comparability.

The reference scenario corresponds to the assumptions used in the baseline analysis. The conservative scenario assumes 20% higher purchase and operating costs, reflecting possible higher maintenance requirements, earlier replacement needs or less favourable market conditions, together with a 20% lower annual healthcare benefit and a higher discount rate of 7%. Conversely, the optimistic scenario assumes 20% lower costs, a 20% higher annual benefit and a discount rate of 3%. These variations are intended to test the robustness of the economic results rather than to represent specific national healthcare systems.

The sensitivity analysis (Table 5) shows that the economic result remains positive across all three scenarios. Under the conservative assumptions, the BCR decreases from 2.75 to 1.83, while the NPV remains positive at approximately €440. Under the optimistic scenario, the BCR increases to 4.13 and the NPV to approximately €1,358. The results therefore suggest that the economic feasibility of the analysed configuration is not entirely dependent on the baseline assumptions. However, the magnitude of the estimated economic benefit varies substantially according to the assumed costs and healthcare-related benefits.

**Table 5:** Sensitivity analysis of the economic assessment under conservative, reference, and optimistic assumptions.

Scenario	Purchase cost (€)	Annual operating cost (€)	Assumed annual benefit (€)	Discount rate	Total 5-year costs (€)	Total 5-year benefits (€)	BCR	NPV (€)
Conservative	288	86.4	264	7%	720	1,320	1.83	440
Reference	240	72	330	5%	600	1,650	2.75	876
Optimistic	192	57.6	396	3%	480	1,980	4.13	1,358

These results should be interpreted as a robustness test of the analytical model rather than as predictions of actual economic returns. In particular, healthcare utilisation, reimbursement arrangements, device prices, and maintenance requirements may differ considerably across European healthcare systems.

To account for uncertainty in the economic assumptions, the reference case was complemented by conservative and optimistic scenarios varying device and operating costs, assumed annual benefits and the discount rate.

### 3.2 Energy consumption

Energy consumption was estimated on the basis of two parameters: the rated power consumption of each device and its assumed operating time. For consistency, a 30-day month was considered. Monthly operating time was calculated from the assumed daily use of each device, and monthly electricity consumption was then estimated according to (3):

Energyconsumption(kWh/month)=Powerconsumption(kW)×Monthlyoperatingtime(h) (3)

The calculation was performed separately for each of the five selected devices. The operating-time assumptions represent indicative patterns of regular home use for the purposes of the analytical assessment rather than directly measured user behaviour. Table 6 reports the assumptions and calculation for each device.

**Table 6:** Estimated monthly energy consumption of the five selected home-monitoring devices based on assumed daily operating time.

Device	Power consumption (W)	Assumed use (min/day)	Monthly operating time (h)	Monthly energy consumption (kWh)
Pulse oximeter	2	10	5.0	0.01
Blood pressure monitor	5	20	10	0.05
Digital thermometer	1	5	2.5	0.0025
Smart ECG monitor	5	20	10	0.05
Blood glucose monitor	3	5	2.5	0.0075
**Total**	**16**	**60**	**30**	**0.12**

Authors’ calculation.

Based on these assumptions, the five devices together consume approximately 0.12 kWh per month. The blood pressure monitor and smart ECG monitor account for the largest shares of the estimated consumption because of their higher rated power and longer assumed operating time. Overall, however, the electricity requirement of the selected home-monitoring devices remains very limited. This result indicates that their operation would have only a marginal impact on overall household electricity demand under the usage assumptions adopted in this study.

Importantly, the low electricity consumption of the devices also confirms that the main rationale for their integration within the SGAM framework is not direct energy saving at device level. Rather, the value of the framework lies primarily in enabling structured interoperability, communication, data exchange and integration with the wider smart-home and healthcare infrastructure.

### 3.3 Potential contribution to elderly well-being

Remote monitoring through IoT-enabled healthcare devices may provide several potential benefits for elderly users beyond the economic and energy dimensions assessed in this study. Continuous access to vital-sign information may support earlier identification of changes in health status, facilitate communication with healthcare providers, and reduce the need for some in-person interactions. In turn, these functionalities may contribute to greater reassurance, autonomy, and continuity of care for older adults, particularly for those managing chronic conditions at home.

However, these potential user-level benefits were not empirically assessed in the present study. No patient surveys, clinical outcomes or validated well-being indicators were collected. Therefore, effects on psychological well-being, perceived security, autonomy, or quality of life should be regarded as expected implications of remote-monitoring systems rather than demonstrated outcomes of the proposed framework. Their assessment would require dedicated studies involving elderly participants and validated health and well-being measures and represents an important direction for future validation of the framework.

## 4 Discussion

SWOT analysis is a method used to identify and assess the strengths, weaknesses, opportunities, and threats of a project (32). This analysis gives a strategic viewpoint that can help better understand the key elements, challenges, benefits, and future potential of integrating smart grids with IoT to improve the reliability and efficiency of healthcare devices and their applications. The SWOT table is described briefly in Table 7.

**Table 7:** Summary of strengths, weaknesses, opportunities, and threats of this model results.

*Strengths*	*Weaknesses*
Comprehensive SGAM framework Real-time data via IoT Portable, low-power, user-friendly devices Affordable and energy-efficient	Limited to elderly users Lack of infrastructure in some regions Data security and privacy concerns Not suitable for all medical conditions
*Opportunities*	*Threats*	
Need to train seniors to adopt extensively. AI can improve accuracy and monitoring. Developing the medical wearables market	Threats to cybersecurity Rapid technological changes and 5G can be a risk. Mishandling due to lack of awareness

The proposed framework is consistent with previous research showing the potential of IoT-enabled devices, gateways, and digital platforms for remote healthcare monitoring. Previous studies have examined IoT healthcare architectures and communication protocols (10), secure gateway-based authentication mechanisms (11), edge-based patient monitoring systems (12), and smart-grid-supported monitoring applications for elderly users (14). Other research has highlighted the role of fog and cloud architectures in supporting distributed healthcare services (15, 19). The contribution of the present study is complementary to these approaches. Rather than proposing a new communication protocol or healthcare device, it adapts the SGAM structure to organize the interaction between heterogeneous home-monitoring devices, communication gateways, healthcare services, and the energy-aware home environment across a common set of interoperability layers, domains, and zones. The economic and energy analyses provide an initial quantitative assessment of this conceptual configuration, but they should be interpreted as scenario-based evaluations rather than as evidence from an operational deployment.

Practical implementation would require particular attention to interoperability, data protection, and regulatory compliance. The use of standards such as IEEE 11073 for personal health-device interoperability and IEC 61850 for energy-related information exchange provides a basis for structuring communication between the healthcare and energy-related components of the framework. However, interoperability alone does not ensure secure operation. Remote monitoring involves the transmission and processing of sensitive health information, making authentication, access control, secure communication, and data governance essential requirements, as also highlighted by previous work on secure IoT healthcare gateways (11). In a European deployment, the processing of personal health data would need to be designed in accordance with the General Data Protection Regulation (GDPR), while medical devices and related software falling within the applicable regulatory scope would need to comply with the relevant requirements of Regulation (EU) 2017/745 on medical devices. SGAM itself does not provide cybersecurity or regulatory compliance mechanisms; rather, it offers an architectural structure within which these requirements can be identified and coordinated across the relevant layers and system components.

User adoption represents a further implementation condition. Although the selected devices were chosen partly because they are portable, widely available, and relatively simple to operate, elderly users differ considerably in digital skills, health status, and ability to interact with connected technologies. Previous research on IoT applications and smart-home healthcare for older adults has similarly identified usability and user interaction as relevant dimensions of technology adoption (18, 27). Consequently, implementation should consider simplified interfaces, appropriate user support and, where necessary, involvement of caregivers or healthcare professionals. The feasibility of the framework may also vary according to the availability and reliability of broadband connectivity, digital healthcare services, smart-home infrastructure, and healthcare-system organization. These differences are particularly relevant when considering replication across European regions and reinforce the need for context-specific validation rather than assuming uniform implementation conditions.

The study presents several limitations that should be considered when interpreting the results. First, the proposed SGAM adaptation is conceptual and has not yet been validated through prototype, simulation, real-world IoT deployment or testing with elderly users and healthcare professionals. Second, the economic assessment relies on secondary European healthcare data, market-based device costs, and scenario assumptions; consequently, the estimated BCR and NPV should not be interpreted as directly transferable to individual national healthcare systems, where reimbursement mechanisms and healthcare costs differ. Third, the energy assessment is based on rated device power and assumed operating times rather than measured household consumption. Fourth, the analysis considers five commonly used home-monitoring devices and therefore does not capture the full diversity of current or future IoT-enabled medical technologies. Future research should consequently validate the adapted SGAM framework in operational home-healthcare environments, assess interoperability and communication performance through prototype implementation, and involve elderly users and healthcare professionals to evaluate usability, acceptance and potential well-being effects using validated indicators. Cross-country testing would also allow the economic and implementation assumptions to be adapted to different European healthcare and infrastructure contexts.

## 5 Conclusion

This study developed an adapted Smart Grid Architecture Model (SGAM) framework for the integration of IoT-enabled healthcare devices in elderly home-based care in Europe. The main conceptual contribution lies in applying the SGAM interoperability structure to organise healthcare devices, users, home gateways, communication networks, digital platforms, healthcare providers, and energy-aware home infrastructure across a common set of layers, domains, and zones. In this context, the relevance of SGAM does not primarily derive from reducing the electricity consumption of individual medical devices, but from providing a structured architecture through which interoperability, communication, data exchange, and system-level requirements can be represented and coordinated.

The conceptual framework was complemented by an analytical assessment of five commonly used home-monitoring devices: a pulse oximeter, blood pressure monitor, digital thermometer, smart ECG monitor, and blood glucose monitor. The economic analysis was based on secondary European healthcare data, market-based device costs, and explicitly defined scenario assumptions. Under the reference scenario, the estimated Benefit-Cost Ratio (BCR) is 2.75 and the Net Present Value (NPV) is positive over the five-year analytical horizon. The sensitivity analysis further shows that the economic result remains positive under the conservative scenario, although the magnitude of the estimated benefits varies substantially according to assumptions concerning device and operating costs, healthcare-related benefits, and the discount rate. These results should therefore be interpreted as indicative evidence of potential economic feasibility rather than as empirically observed or directly transferable returns across European healthcare systems.

The revised energy assessment estimates a combined electricity consumption of approximately 0.12 kWh per month for the five selected devices under the assumed usage pattern. This confirms that their direct contribution to household electricity demand is marginal. The result also reinforces the interpretation of SGAM primarily as an interoperability and system-integration framework rather than as a mechanism for achieving substantial device-level energy savings.

The proposed framework may also support broader benefits related to continuity of care, access to health information, reassurance, and autonomy for elderly users. However, these outcomes were not empirically assessed in the present study and should therefore be understood as potential implications rather than demonstrated effects. The study is subject to further limitations, including the absence of prototype deployment or patient testing, reliance on secondary data and market-based assumptions, the use of estimated rather than measured energy consumption, and the consideration of a limited set of home-monitoring devices. Future research should consequently focus on validating the adapted SGAM framework in operational home-healthcare environments, testing interoperability and communication performance, and involving elderly users and healthcare professionals to assess usability, acceptance, and well-being outcomes through validated indicators. Cross-country validation would also allow the economic and implementation assumptions to be adapted to different European healthcare, reimbursement, and infrastructure contexts.

## Data Availability

The original contributions presented in the study are included in the article/Supplementary Material, further inquiries can be directed to the corresponding author.
